# Profound Depletion of HIV-1 Transcription in Patients Initiating Antiretroviral Therapy during Acute Infection

**DOI:** 10.1371/journal.pone.0013310

**Published:** 2010-10-12

**Authors:** Adrian Schmid, Sara Gianella, Viktor von Wyl, Karin J. Metzner, Alexandra U. Scherrer, Barbara Niederöst, Claudia F. Althaus, Philip Rieder, Christina Grube, Beda Joos, Rainer Weber, Marek Fischer, Huldrych F. Günthard

**Affiliations:** Division of Infectious Diseases and Hospital Epidemiology, University Hospital Zürich, University of Zürich, Zürich, Switzerland; University of California San Francisco, United States of America

## Abstract

**Background:**

Although combination antiretroviral therapy (cART) initiated in the acute phase of HIV-1 infection may prevent expansion of the latent reservoir, its benefits remain controversial. In the current study, HIV-1 RNA transcription patterns in peripheral blood mononuclear cells (PBMC) were monitored during acute cART to assess the effect of early treatment on cellular viral reservoirs.

**Methodology/Principal Findings:**

Acutely HIV-1 infected patients (n = 24) were treated within 3–15 weeks after infection. Patients elected to cease treatment after ≥1 year of therapy. HIV-1 DNA (vDNA), HIV-1 RNA species expressed both in latently and productively infected cells, unspliced (UsRNA), multiply spliced (MsRNA-tatrev; MsRNA-nef), and PBMC-associated extracellular virion RNA (vRex), expressed specifically by productively infected cells, were quantified in PBMC by patient matched real-time PCR prior, during and post cART. In a matched control-group of patients on successful cART started during chronic infection (n = 15), UsRNA in PBMC and vDNA were measured cross-sectionally. In contrast to previous reports, PBMC-associated HIV-1 RNAs declined to predominantly undetectable levels on cART. After cART cessation, UsRNA, vRex, and MsRNA-tatrev rebounded to levels not significantly different to those at baseline (p>0.1). In contrast, MsRNA-nef remained significantly lower as compared to pretreatment (p = 0.015). UsRNA expressed at the highest levels of all viral RNAs, was detectable on cART in 42% of patients with cART initiated during acute infection as opposed to 87% of patients on cART initiated during chronic infection (Fisher's exact test; p = 0.008). Accordingly, UsRNA levels were 105–fold lower in the acute as compared to the chronic group.

**Conclusion:**

Early intervention resulted in profound depletion of PBMC expressing HIV-1 RNA. This is contrary to chronically infected patients who predominantly showed continuous UsRNA expression on cART. Thus, antiretroviral treatment initiated during the acute phase of infection prevented establishment or expansion of long-lived transcriptionally active viral cellular reservoirs in peripheral blood.

## Introduction

Current combination antiretroviral therapy (cART), despite its potency in suppressing active viral replication [1], [2] and its power in reducing mortality and morbidity of HIV-1 infection [3], [4], has not resulted in eradication or induction of treatment-free periods of remission of HIV-1 replication [5], [6], [7]. A pool of HIV-1 infected long-lived latently infected memory T-lymphocytes has been reported to be the major reservoir that confers HIV-1 infection resilient to eradication [8], [9], [10]. The frequency of latently infected cells was reported to range from 1 to 20 cells per 10^6^ resting CD4^+^ T-cells [9]. However, as these assays rely on technically demanding ex-vivo outgrowth assays, their results likely underestimate the size of the latent reservoir. Recent studies used PCR-based methods, in which latency had been defined as active viral transcription in the absence of viral progeny production. The resulting estimates of the size of the peripheral blood latent reservoir were at least 5–10 times higher in resting CD4^+^ T-cells [11] as well as in total PBMC [12] when compared to viral outgrowth assays [9]. Apart from cells in peripheral blood, other sites such as lymphoid tissues [13], [14], [15], the gastrointestinal tract [16], [17], [18], [19], the brain [20], and the genital tract [21], have been reported to contribute to latent viral sanctuaries.

Due to the low expression levels of viral RNA in latently infected cells [12], [14], [22], [23], [24] and viral antigens, the latent viral reservoir is greatly inaccessible for adaptive and innate immune defenses. It has been proposed that local bursts of viral replication are initiated by immune activation in response to specific antigens [25], [26], [27] or due to random self-activation by the viral transactivator Tat [28]. In the presence of potent cART, such bursts will be dead-ended, because the viral particles produced may not initiate new rounds of infection. Conversely, after cessation of cART, this process will lead to rapid viral rebound [29] initiated stochastically from single or oligoclonal archival proviruses [30].

Nevertheless, the canon that HIV-1 replication, as measured by levels of plasma viremia, will inevitably proceed in the absence of cART [31], [32] or recur soon after its cessation [29], [33] has been challenged by important exceptions. Due to strong and specific immunological responses, so called elite-controllers contain viral replication to levels below a clinically relevant threshold (<50 copies/ml) in the absence of cART (reviewed in [34]). Furthermore, in some instances treatment initiated during the acute or early phases of HIV-1 infection had resulted in control of viremia after treatment cessation [35], [36], [37], [38]. These anecdotal cases have been used as precedents supporting early treatment. In addition, two rationales have been proposed to affirm possible benefits of early treatment of HIV-1, despite considerable side effects [39], [40], [41] as well as costs [42], [43], which need to be considered in relation to decades of expected treatment time.

Paul Ehrlich's paradigm to “hit early and hard” in treatment of infectious disease [44] to limit spread of an infectious pathogen and to contain its population size, toxicity, and its potential to escape immunological and chemotherapeutic/medical control [45] is still valid and accepted. This concept was substantiated for HIV-1 by Strain et al., who showed that the size of latent reservoirs was smaller in patients with treatment initiation in the acute phase than in those who initiated cART during chronic infection [46]. In addition to that, the notion that acute HIV-1 infection is generally associated with oligoclonal viral quasispecies of low phylogenetic diversity [47], [48], led to the hypothesis that by early treatment viral escape to specific HIV-1 antibody- and cytotoxic T-cell responses may be avoided or deferred resulting in clinical benefit and/or virological control of the virus. This concept was corroborated by a study showing that transmission of multiple quasispecies was associated with faster disease progression than mono- or oligoclonal primary infection [49]. Low viral diversity has also been shown to be associated with modest but significant control of viremia in chronic infection [50]. Moreover, superinfection can lead to the loss of control of viremia [51], [52], [53], [54], [55], [56].

To elucidate these issues in the current study, intra- and extracellular viral dynamics were assessed before, during, and after treatment of acute HIV-1 infection. For this purpose HIV-1 RNA transcription patterns in PBMC and levels of plasma viremia were monitored. Comparison with a group of patients, who had initiated cART during chronic infection, was performed cross-sectionally. Unexpectedly, cell-associated viral RNA burden in PBMC became mostly undetectable in the acutely infected patient group. The levels of UsRNA, the most abundant cellular HIV-1 RNA *in vivo*, were at least one order of magnitude lower during cART initiated in acute than in chronic infection. These results strongly indicate that patients initiating cART in the acute phase of infection may attain a substantial virological benefit from early intervention.

## Results

### Kinetics of viral RNA levels in response to treatment and cessation of cART

**Figure 1:**
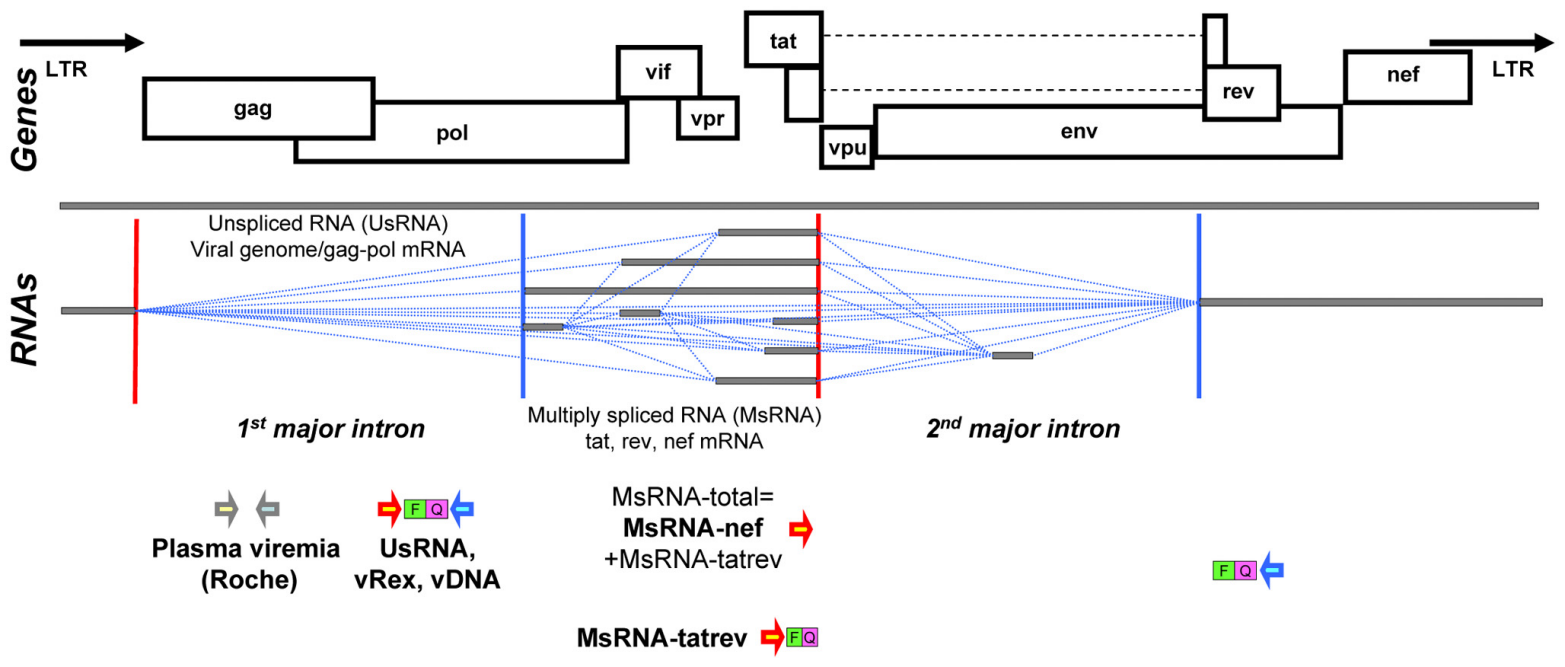
Map of HIV-1 amplica for qPCR assays. Splice acceptors and donor sites are shown by red and blue vertical lines, respectively. Exons are depicted in grey bars. Blue dotted lines show documented or predicted splice events. Sense primers used in qPCR assays are depicted by red arrows antisense/cDNA primers by blue arrows. Fluorescent hydrolysis probes [58] (FH-probes/TaqMan®-probes) are shown by green/pink bars. Assays for MsRNAs share a common antisense/cDNA primer and in some instances, when a probe for MsRNA-tatrev was not available, a common FH-probe 3′ of the splice-acceptor of the 2^nd^ major intron. Note that this map does not show mRNAs encoding Env because these transcripts were not assessed in the current study.

Acutely HIV-1 infected patients (n = 24) were treated with cART within 3–15 weeks after infection. Plasma viremia was monitored using standard diagnostic quantitative PCR-assays (qPCR) and PBMC-associated HIV-1 nucleic acids (figure 1) were quantified by patient matched qPCR [11], [12], [57], [58]. HIV-1 DNA (vDNA) was assessed as a measure of total infected cells, comprising latently and productively infected cells as well as cells harboring defective genomes.

To investigate the activity of these HIV-1 infected cells, distinct PBMC-associated viral RNA species (vRNAs) were quantified: unspliced RNA (UsRNA) and 2 types of multiply spliced encoding tat and rev (MsRNA-tatrev) or nef (MsRNA-nef). These RNAs were found to be expressed both in latently [12], [23], [59] and productively infected cells, however, at much higher levels in the latter cell population. As a marker for productively infected cells, PBMC-associated extracellular virion RNA (vRex) was measured [11], [12], [14], [22], [57].

**Figure 2:**
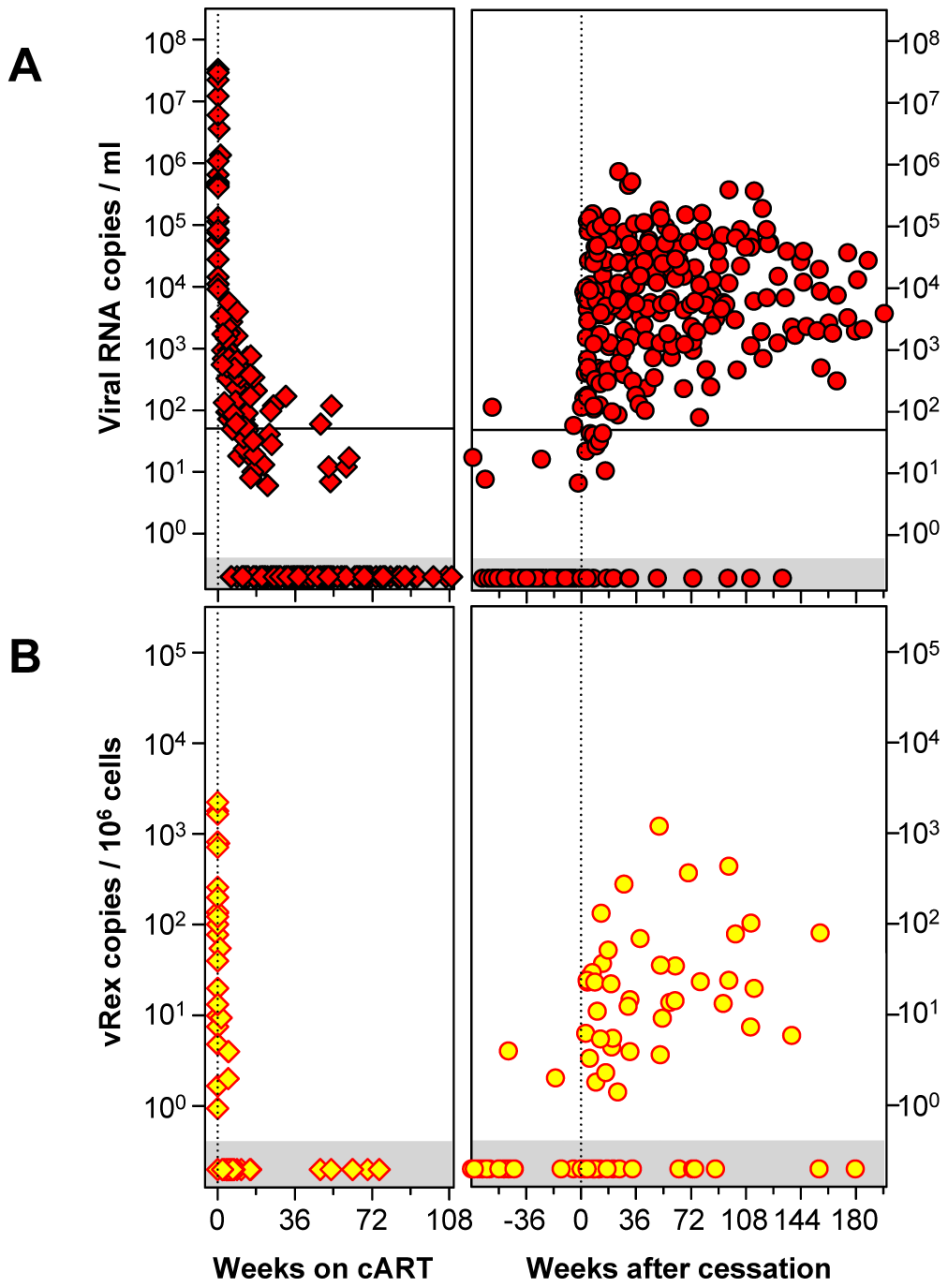
Longitudinal course of HIV-1 virion production. Levels of plasma viremia (**A**) and of PBMC-associated virions, vRex (**B**) of 24 acutely infected patients, were plotted against time after initiation of cART (0 weeks, left panels) or against time after cART cessation (defined as 0 weeks in the right panels). Note that some data points are depicted in the left panels are also shown in the right panels to facilitate visualization of the effects of cART cessation. The amount of vRex measurements is lower, because cell sampling was done less frequently than plasma HIV-RNA was measured. Data within the grey horizontal areas show PCR-negative samples below the detection limit of the assays applied. The black line in panel A depicts the clinically used threshold for plasma viremia of 50 RNA copies/ml.

**Figure 3:**
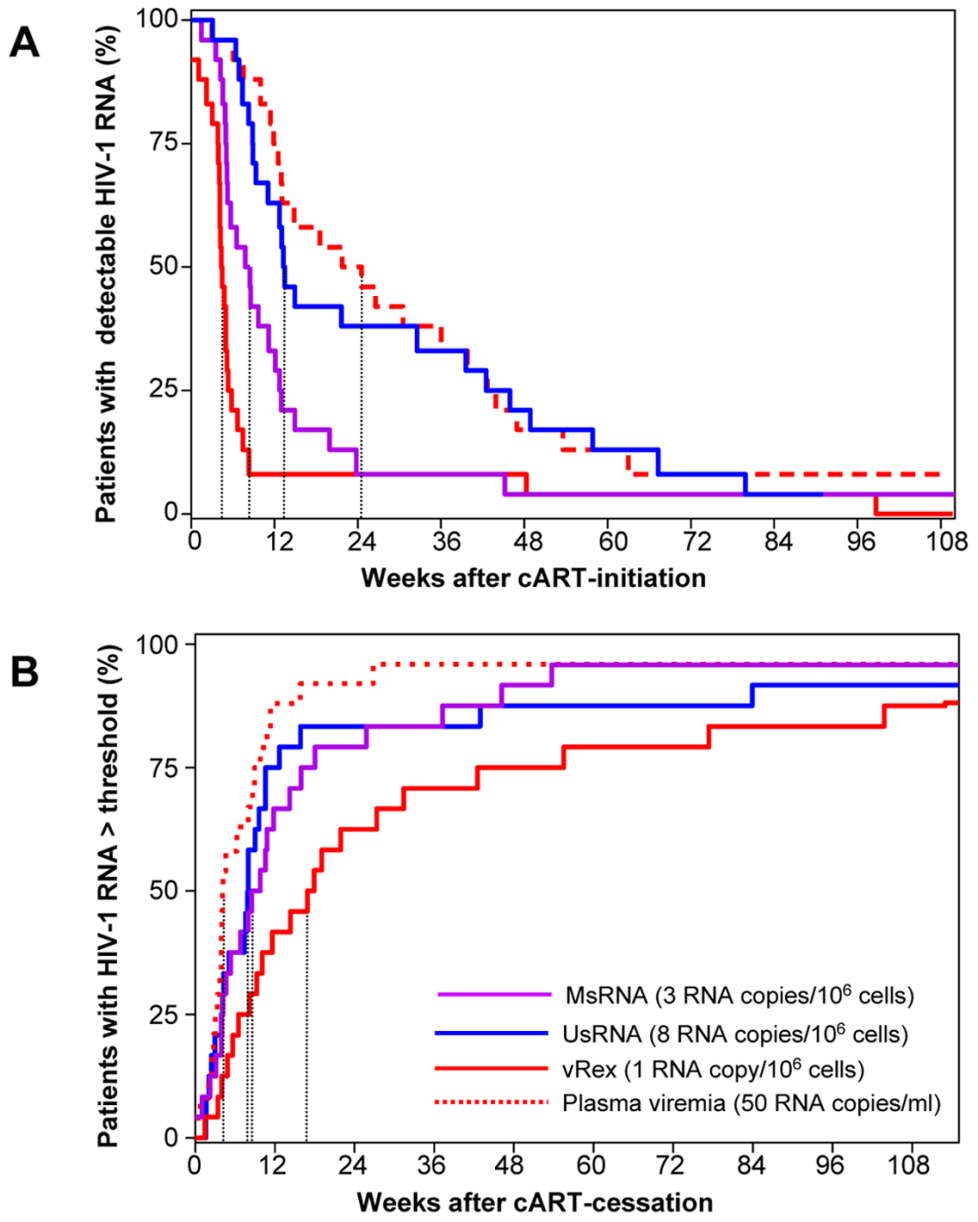
Empirical distribution of times to virological end-points. Time to event analysis showing the percentage of patients before reaching their first PCR-negative measurement after initiation of cART (**A**) or reaching measurable viral RNA levels above a defined threshold after cessation of cART (**B**). Thresholds are indicated in parentheses in panel B and were defined as approximately 3-fold elevation over mean on cART levels for cellular HIV-1 RNAs. Threshold of plasma viremia was set at 50 copies/ml. On average vRex (red lines), MsRNAs (magenta), UsRNA (blue) and plasma viremia (red broken lines) dropped to undetectable levels 4.5, 8.4, 13.5, and 24.6 weeks after initiation of cART, respectively (A) and rebounded within 17.9, 9.8, 8.0, and 4.2 weeks after treatment cessation (B), (dotted lines). Note that for analysis of plasma viremia only time-points were considered for which also UsRNA and MsRNA measurements had been available.

Initiation of cART resulted in a decay of viral RNA production (figure 2A/B left panel). Whereas plasma viremia followed kinetics which were consistent with a 2–3 phasic exponential decay, vRex dropped to undetectable levels almost instantly after initiation of cART. Decay of UsRNA and MsRNAs was intermediate between that of plasma viremia and vRex (data not shown). Upon cessation of cART, rebound of HIV-1 nucleic acids occurred quickly in plasma (figure 2A, right panel), but was apparently delayed in PBMC (figure 2B, right panel, figure 3B).

### Time to event analysis of HIV-1 RNA detection during and after cART

To corroborate these observations and to extend them to UsRNA and MsRNAs, empirical distributions of the proportions of patients with detectable viral RNAs after initiation of cART were plotted (figure 3A). Similarly, distribution of the proportion of patients experiencing viral rebound after therapy cessation was addressed (figure 3B). Upon initiation of cART, disappearance of vRex took a median time of 4.5 weeks (quartiles 4, 6 weeks). Taking into account that the second sample following baseline in the current study was obtained at a median time of 4.4 weeks (quartiles 4, 5 weeks) depletion of vRex occurred instantaneously after cART initiation. MsRNAs declined significantly later to undetectable levels (Mann-Whitney, p = 0.002), namely within a median time of 8.4 weeks (quartiles 5, 13 weeks). The next parameter to reach its detection limit with a significant delay compared to MsRNA (Mann-Whitney, p = 0.003) was UsRNA with a median time to the first undetectable measurement of 13.5 weeks (quartiles 9, 47 weeks). Finally, plasma viremia dropped to undetectable levels within a median time of 24.6 weeks (quartiles 12, 44 weeks), which was not significantly different to the decay of UsRNA (Mann-Whitney, p = 0.52). In general, after detection of the first undetectable HIV RNA value, all RNA species measured remained undetectable at almost all timepoints measured during treatment.

After cessation of cART, plasma viremia reached levels above the threshold of 50 copies/ml in a median time of 4.2 weeks (quartiles 3, 10 weeks). Rebound of UsRNA and MsRNA proceeded with some delay without reaching statistical difference (Mann-Whitney, p = 0.30 and p = 0.07, respectively): Median time to rebound was 8.0 weeks (quartiles 4, 12 weeks) and 9.8 weeks (quartiles 4, 19 weeks) for UsRNA and MsRNA, respectively. Lastly, within a median time of 17.9 weeks (quartiles 8, 56 weeks) vRex rose to detectable levels. Its rebound was statistically different from that of plasma viremia and of UsRNA (Mann-Whitney, p = 0.007 and 0.02, respectively) but not from MsRNA (Mann-Whitney, p = 0.06). It has to be noted that vRex was measured less frequently than plasma or PBMC viremia. Therefore the median time to rebound may have been overestimated.

### Magnitude of viral nucleic acid levels at baseline and post-cessation

To investigate whether cART initiated in acute infection had an impact beyond its cessation, the levels of HIV-1 nucleic acids at baseline and after treatment cessation were compared. Maxima were used to represent post-cessation HIV-1 nucleic acid levels, to account for the variability among the different parameters regarding the time to reach stable set-points.

**Figure 4:**
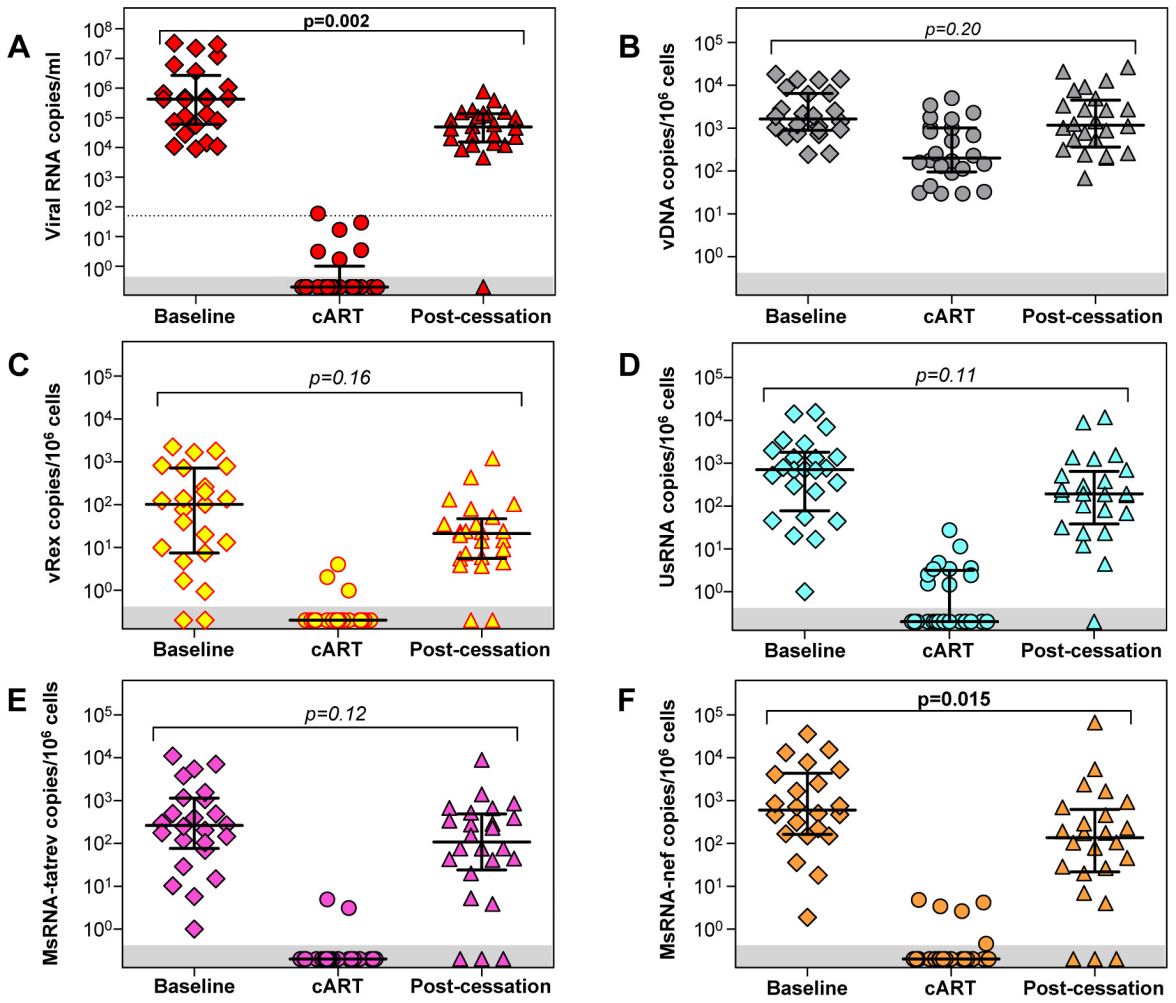
Virological parameters before, during, and after cART. Viral nucleic acid levels at baseline (diamonds), and average values during the time windows defined as on cART (circles, vRex ≥2 weeks, plasma viremia, PBMC-associated UsRNA and MsRNAs ≥24 weeks) and post-cessation maximal values (triangles). Data-points within the grey horizontal areas show PCR-negative samples below the detection limit of the assays applied. P-values within panels show significance levels (Wilcoxon signed rank test) between baseline and post-cessation. Bars show medians and quartiles. The dotted line in panel A depicts the clinically used threshold for plasma viremia of 50 RNA copies/ml.

Plasma viremia (figure 4A) was significantly reduced after treatment cessation as compared to baseline (Wilcoxon signed rank test, p = 0.002). This reduction can be attributed in part to the early treatment effect of cART (Gianella, von Wyl *et al*., unpublished data), but also to a naturally occurring “first peak” of viremia observed in acute HIV-1 infection [60]. We observed a less pronounced impact of early cART on cellular viral nucleic acid levels after treatment cessation. Only MsRNA-nef showed persistent significant reduction after cART (Wilcoxon signed rank test, p = 0.02), whereas the remaining parameters, vDNA, vRex, UsRNA, and MsRNA-tatrev (figure 4B–E) tended to be lower after treatment stop compared to baseline but without reaching statistical significance (Wilcoxon signed rank test, p>0.10).

### Viral reservoirs during cART

As opposed to baseline and post-cessation viral nucleic acid levels, which were represented by single measurements, on-cART levels were estimated by calculating means. Taking into account the rapid decay of vRex, the mean of samples at time-points ≥2 weeks after initiation of treatment was used. For all other parameters measurements taken at time points ≥24 weeks were used for calculation of mean values. The rationale for choosing this time-window was that the parameter with the slowest decay kinetics, plasma viremia, reached undetectable levels within a median time of around 24 weeks as shown in figure 3A. Accordingly, the resulting mean values for plasma viremia on cART were well below the clinically used threshold of 50 copies/ml (figure 4A), except for one patient (#133; 59 copies/ml).

HIV-1 DNA persisted at a level of 823±253 copies/10^6^ PBMC (mean±sem) during cART initiated in acute infection. However, proviruses in these infected cells were transcriptionally almost completely silent. Expression of MsRNAs and vRex was mostly undetectable and attenuated to mean levels <1 copy/10^6^ PBMC (MsRNA-tatrev: 0.34±0.24, MsRNA-nef: 0.65±0.30, vRex: 0.29±0.19; mean±sem). Only expression of UsRNA was occasionally detected at a mean level of 2.6±1.2 copies/10^6^ PBMC. The absence of vRex during cART observed in the current study was similar to findings of previous studies [11], [12], [14], [22] indicating a fast and complete loss of productively infected cells as a consequence of cART. However, the almost complete depletion of MsRNA and the profound reduction of UsRNA were highly unexpected, as we and others have shown their presence at measurable levels in latently infected cells of HIV-1 infected patients on cART [11], [12], [59].

### Comparison of the effects of cART initiated in primary versus chronic infection

**Figure 5:**
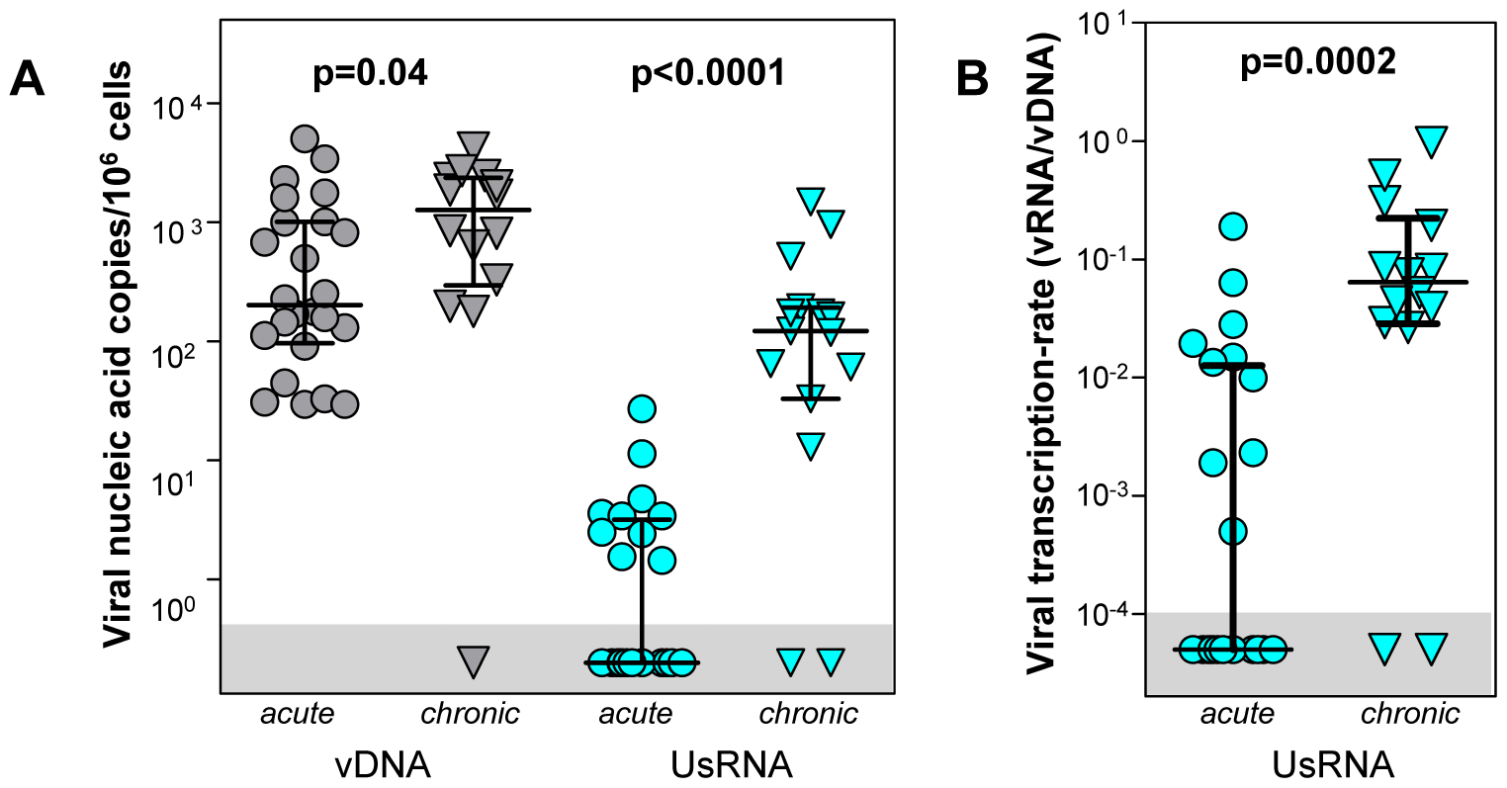
Viral nucleic acid expression in acute and chronic patients during cART. Mean levels of vDNA (grey symbols) and UsRNA (blue symbols) (**A**) or viral transcription rates (**B**) of patients that had initiated cART during acute infection (acute, n = 24, circles) were assessed during the on-cART time window (≥24 weeks) and compared to single measurements in patients that had initiated cART during chronic infection (chronic, n = 15, triangles, median treatment time 50 weeks, range 36–183, see Table S1 for details). Data-points within the grey horizontal areas show PCR-negative samples below the detection limit of the assays applied. P-values within panels show significance levels (Mann-Whitney test) between the acute and the chronic group. Bars show medians and quartiles.

To investigate whether the observed depletion of viral transcription could be attributed to the early initiation of cART, PBMC obtained from patients, who had initiated antiretroviral therapy in the chronic phase of HIV-1 infection, were assessed for the presence of viral RNA in PBMC. Latently infected cells expressing solely UsRNA had been shown to persist during cART at the highest frequency among all cell classes expressing viral RNA and UsRNA had been consistently detected at the highest levels during cART in previous studies [11], [12], [57], [61]. Therefore, UsRNA and vDNA were quantified in patients in whom cART had been initiated during chronic infection (n = 15, for patient characteristics see Table S1) using identical conditions as for the acute patient group, including RNA extraction, use of patient matched primers, and PCR input volumes. Levels of plasma viremia in the chronic control group were <50 copies/ml (mean: 10 copies/ml, range 0–28) and treatment had lasted for a median time of 50 weeks (36–183 weeks). In this group of patients, 86% of specimens were positive for UsRNA, which is significantly higher than the percentage of patients in the acute group showing any UsRNA positive PCR on cART (42%, Fisher's exact test, p = 0.008). Furthermore, the average level of UsRNA in the chronic control group (mean = 272 copies/10^6^ cells, range 0–1486) was 105-fold higher than in the acute group (Mann-Whitney, p<0.0001, figure 5A).

Levels of vDNA were significantly elevated (Mann-Whitney, p = 0.04) in the chronic group as compared to the patients treated during acute infection. To adjust for this difference in the total number of infected cells, the average viral transcriptional rate i.e., the ratio of viral RNA to vDNA, was calculated [11]. Similarly as the absolute levels of UsRNA, viral transcriptional rates were elevated with high statistical significance (12-fold, Mann-Whitney, p = 0.0002) in the chronic group as compared to patients in the acute group (figure 5B).

## Discussion

The main aims and rationales for antiretroviral therapy in acute HIV-1 infection are confinement of genetic diversity of viral quasispecies to prevent escape from adaptive immunity, restriction of CD4^+^ T-cell loss, preservation of immune functions, and inhibition of initial viral spread throughout the possible sites of replication. Despite considerable efforts from various research groups, to date evidence from clinical studies in humans to empirically substantiate these concepts remains limited. Here we performed a longitudinal in-depth analysis using patient matched highly sensitive qPCR assays to detect different HIV-1 gene trancripts to assess effects of early treatment on cellular reservoirs of HIV-1 in peripheral blood.

HIV-1 transcription was monitored longitudinally before, during, and after cessation of treatment of acute HIV-1 infection in well characterized patients [60], [62]. This analytical approach was applied because numerous studies revealed persistence of cellular HIV-1 RNA for years in patients on cART despite suppression of plasma viremia [14], [15], [17], [57], [61], [63], [64], [65]. By choosing a patient matched approach for qPCR with single copy sensitivity, it was anticipated that not only levels of UsRNA, but also the less abundant multiply spliced RNAs could be quantified and used to assess the viral transcription pattern of the PBMC reservoir of HIV-1 during cART [11], [12], [57].

The major finding of this study was that early cART initiated during acute HIV-1 infection significantly depleted the number of transciptionally active proviruses by an order of magnitude when compared to levels detected in patients treated during chronic infection.

Specifically, productively infected PBMC, hallmarked by expression of cell attached virions (vRex) were depleted within a median time of 4.5 weeks after cART initiation. The remaining HIV-1^+^ PBMC, expressing solely intracellular viral RNA, can therefore be viewed as latently infected [11], [12], [14]. These cells faced depletion within a median time of 24 weeks. This resulted in >100-fold reduction of viral transcription levels and at least 10-fold lower average viral transcriptional rates as compared to patients who had started cART during chronic infection. It is unlikely that the strong reduction of viral transcription in PBMC in primary HIV infected patients was due to the potency of treatment rather than to timing of antiretroviral therapy because the control group of chronically infected patients received similar treatment.

Decay of HIV-1^+^ cells in response to treatment occurred in a significantly staggered mode according to their pattern of viral RNA expression and life-span as previously described [12]. Productively infected cells expressing vRex vanished almost instantly after initiation of treatment, then cells expressing MsRNA followed with a delay of about 4 weeks, ultimately a further 5 weeks later, cells expressing solely UsRNA pursued. Thus, even HIV-1 RNA expressing cells with the longest life spans [12] approached depletion.

These findings imply that early cART led to the clearance of long-lived cells harboring transcriptionally active latent proviruses. In an early study, Markowitz and colleagues showed persistence of viral transcription in PBMC when antiretroviral therapy was initiated within 90 days after onset of primary symptoms [17]. This discrepancy can most likely be explained by a 5–6 week earlier initiation of therapy in the present study. Of note, our findings are in agreement with the observations of Strain and colleagues [46]. In their study, using viral outgrowth assays of resting CD4^+^ T-lymphocytes, a cohort of patients on early cART showed lower levels of latently infected cells than a control group with deferred cART initiation. In addition, two small studies in humans and in the SIV model also suggest that HIV-1 suppression as measured by unspliced HIV-1, respectively SIV RNA can be reduced by treatment during primary HIV infection in the gut associated lymphoid tissue [66], [67].

After cessation of cART, virological parameters reappeared inverted to their decay. UsRNA rose to significant levels after 8 weeks, followed two weeks later by MsRNA and finally vRex reappeared with a significant delay. The observations that viral rebound in plasma preceded that of cellular viral RNAs and plasma viremia was the last parameter to decay during cART, implies that PBMC can be viewed as independent compartment to some degree separate from the source of virus appearing in plasma. These dynamics potentially suggest that repopulation of the PBMC compartment of rebounding transcriptionally active PBMC was most likely due to “*de novo*” infection of PBMC by plasma virions rather than by reactivation of latently infected PBMC. The rebounding virus most likely originates from distinct compartments such as the secondary lymphoid organs (e.g., gut associated lymphoid tissue (GALT), lymph nodes, spleen), the cerebral nervous system, the kidneys, or the genital tract. Very recent data by Yukl *et al.* [19] suggests that most likely more than 83% of all HIV-1 infected cells under therapy are to be found in the GALT and that the RNA/DNA ratio on treatment tends to be higher in this compartment than in the peripheral blood. Furthermore, GALT is continuously exposed to bacterial antigenic stimuli from gut commensals. In aggregate, the high HIV burden together with continuous antigenic stimulation makes this large reservoir a prominent candidate as a source for the rebounding virus. Of note, the RNA/DNA ratios of GALT and PBMC in the study by Yukl was measured with exactly the same methods as in our study.

The fact that post-cessation control of viral RNA expression was achieved in several patients during the post treatment observation period supports this hypothesis: Plasma viremia was undetectable in patient #72, control of UsRNA expression was experienced in patient #25, control of MsRNA expression was achieved by patients #25, #56, and #92, and control of vRex was experienced by patients #72 and #99.

The observation that MsRNA-nef levels, a parameter reported to be associated with viral latency [57], [68] were significantly reduced as compared to baseline further indicates that the latent reservoir was reduced by initiation of cART during acute infection. Conversely, it cannot be fully excluded that the decreased levels of MsRNA-nef after therapy cessation may be explained by the “first peak” of viral replication in acute HIV-1 infection and its subsequent spontaneous decay independent of cART [69].

Taken together, our data demonstrate a profound virologic effect of treatment initiation during acute HIV-1 infection. The unprecedented finding that residual viral transcription was almost completely depleted, suggests that the latent reservoir of HIV-1 during cART can be reduced by early intervention and that proof of concept studies aiming at HIV-1 eradication or remission of HIV-1 replication should be initiated in patients during acute infection.

## Materials and Methods

### Ethics Statement

In accordance with the guidelines of the Ethics Committee of the University Hospital of Zurich, written informed consent was obtained from all participants of the studies analyzed: the Zurich Primary HIV Study (ZPHI), the Swiss Spanish Intermittent Therapy Trial (SSITT), and the Zurich HIV-1 Transcription Study.

### Patients and specimens

#### Patients with treatment initiated in the acute phase of HIV-1 infection (acute group)

Patients (n = 24; Table S1) included in our study were enrolled in the Zurich Primary HIV Study (ZPHI, http://clinicaltrials.gov, NCT00537966) [62] a substudy of the Swiss HIV Cohort Study (SHCS). All 24 subjects had a documented acute or recent primary HIV-1 infection at presentation. Estimation of time after infection was as described by Rieder et al [60]. Patients were included in the analysis only if sequences were available to match primers in *pol, tat*, and *env*.

Blood samples were collected at the day of the very first cART application ( = baseline sample), longitudinally during treatment, and after treatment cessation. EDTA-blood samples were collected and separated into cells and plasma according to standard procedures [22]. Cellular material was split into aliquots, each comprising 2 million cells, and finally stored at −80°C as dry cell pellets. Sampling of specimens for analysis of plasma viremia and cellular nucleic acids was performed as described in Methods S1. All patients were extensively tested for genotypic drug resistance: Only one patient showed the D67N mutation at baseline in the routine genotyping test and in addition, one patient showed 0.02% K103N and one 2.4% M184V harboring minority species [62].

#### Patients with treatment initiated in the chronic phase of HIV-1 infection (chronic group)

PBMC samples for a cross-sectional control group of 15 patients treated in the chronic phase after seroconversion were obtained from the Swiss Spanish Intermittent Therapy Trial (SSITT) [33] at the last time-point before treatment cessation. In addition, specimens collected for The Zurich HIV-1 Transcription Study (INFZ VTA 02.00 [12]) were analyzed (n = 9). As in the acute group, patients in the chronic group participated also in the Swiss HIV Cohort Study (SHCS) and HIV-1 *pol*-sequences were available from the SHCS genotypic drug resistance database [70]. Patient characteristics of the chronic group are listed in Table S1.

### Nucleic acid extraction/preparation

Total PBMC RNA was extracted with the RNeasy extraction kit on the ‘QIAcube’ extraction device (Qiagen Hombrechtikon, Switzerland) using an initial volume of 0.35 ml for cell lysis, DNAse I digestion on the extraction column and a final elution volume of 0.1 ml.

Selective isolation of PBMC-associated extracellular, virion-encapsidated genomic HIV-RNA, vRex, free of intracellular nucleic acid, was performed as described [14], [22], [71] using an elution volume of 0.1 ml. Preparation of protease digested total cell-lysates for DNA quantification was performed according to Christopherson et al. [72] with minor modifications [11].

### Design of HIV-1 patient matched qPCR

To ensure accurate quantification a patient matched approach reaching single copy sensitivity was used for qPCR measurements of RNA and vDNA [11], [12], [57], [58] using specifically designed fluorescent hydrolysis probes [58]. The location of qPCR primers and probes is shown in figure 1; sequences are outlined in detail in Table S2 and Methods S1. Individual sequence information to adjust qPCR assays for UsRNA, vRex, and vDNA, was obtained from the SHCS genotypic drug resistance database (HIV-1 *pol*-sequences) [70].

To obtain sequence information to match qPCR primers and probes for MsRNAs of predominant quasispecies, two regions were amplified from each patients total DNA by a nested PCR scanning approach as described [11] (positions 5833-6152 and 7992-8567 in the HXB2 genome, GenBank accession number K03455).

As previously described [11], [12], [57] two different assays for MsRNA were performed: MsRNA-total and MsRNA-tatrev from which the MsRNA-nef was calculated using the difference in copy numbers between the two measurements. To avoid redundancy, MsRNA-total was not reported. Thus, only MsRNA-tatrev and MsRNA-nef are shown in the current data-set and used for statistical calculations. In 11% of measurements, it occurred that copy numbers of MsRNA-total were nominally lower or equal to MsRNA-tatrev. Calculation of MsRNA-nef was considered as not applicable in these cases.

### Quantitative real-time PCR measurements

Assays for RT-qPCR were performed as described [57], [73] using an ABI 7500 real-time thermocycler (Applied Biosystems, Rotkreuz, Switzerland) with 0.005 µM ROX as passive reference, 0.2 µM fluorescent probe and 5 µl RNA as template in a final volume of 35 µl aqueous phase plus 15 µl paraffin. Quantification of vDNA was performed as described by Althaus *et al.* [58] in an IQ5 real-time thermocycler (Biorad, Basel, Switzerland) in a volume of 60 µl with 10 µl DNA template using HotStarTaq master mix (QIAGEN, Hilden, Germany) supplemented with PCR primers (1 µM each), probe (0.3 µM), and additional MgCl_2_ (1.5 µM) by incubation for 15 min at 95°C and 60 cycles of 10 s at 95°C, 5 s at 55°C and 40 s at 60°C.

### Calculation of HIV-1 nucleic acid copy numbers from qPCR data

Patient-nucleic acids were directly used to generate HIV-1 standard-curves. To obtain standard curves with maximal dynamic range, baseline specimens were used as standards for vDNA-measurements. Post-cessation time points with high plasma viremia and high vDNA were chosen. Standards were prepared as serial 3-fold dilutions in quadruplicates. Specimens other than the ones chosen as standards were measured in duplicate.

Linear regression used in standard real-time PCR analysis was applied for calculation of HIV-1 copy numbers:
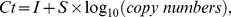
(1)where *I* is the Y-axis intercept of the regression line, depicting the point where Ct equals one copy ( = 10^0^) and *S* is its slope. *I* and *S* were determined as described in Methods S1. In cases where only one duplicate HIV-1 PCR was positive and/or calculation resulted in less than 1 nominal copy number per reaction, HIV-1 nucleic acid copy numbers were censored to 1 copy per PCR [11], [12].

### Normalization to cellular input

To control for input and/or differences in qPCR efficiencies in RNA PCR the expression of glyceraldehyde 3-phosphate dehydrogenase (GAPDH) against a standard dilution series of in vitro transcribed GAPDH RNA was measured in duplicates as described previously [73] and in Methods S1. Linear regression, as applied by the ABI7500 software, was used to evaluate GAPDH measurements. Mean GAPDH-RNA copies per 10^6^ cell equivalents were empirically determined in PBMC specimens with known cell numbers (2×10^6^ cells sample, n = 310) resulting in a conversion factor of 2.85×10^7^ copies GAPDH-RNA/10^6^ PBMC.

Cellular input for DNA was quantified by beta-actin PCR using primers and probe described in Methods S1. As quantification standard for cellular input, 15 million PBMC were lysed in 1 ml cell lysis buffer, serial two-fold dilutions were measured and cellular input per PCR was calculated by linear regression using the software provided with the IQ5-system. Both for vDNA and viral RNA, the mean of duplicate PCR measurements was normalized to cellular input and finally expressed as HIV-1 nucleic acid copies per 10^6^ PBMC.

### Statistical analysis

GraphPad Prism 5.0 software (GraphPad Software, San Diego, CA) was used for statistical analyses. P<0.05 was considered as the level of significance. Mann-Whitney testing was applied unless otherwise indicated for group comparisons. Fisher's exact test was used in analysis of contingency tables. No adjustment for multiple testing was applied.

## Supporting Information

Table S1 Characteristics of patients.
(0.01 MB PDF)

Table S2 Oligoucleotides used for patient-specific qPCR.
(0.10 MB PDF)

Methods S1 Supplementary methods.
(0.05 MB PDF)
