# Supplementary material for: Profound Depletion of HIV-1 Transcription in Patients Initiating Antiretroviral Therapy during Acute Infection
Source: PLoS One. 2010 Oct 12;5(10):e13310. doi: 10.1371/journal.pone.0013310 (PMC2953504; doi:10.1371/journal.pone.0013310)
Supplement: Table S1 — Characteristics of patients. (0.01 MB PDF) [file pone.0013310.s001.pdf]

TABLE S1-A: Characteristics of patients in the “*acute*” group

ID	Treatment(s) [A]	Infection to initiation of cART (weeks)	Duration of cART (weeks)	Baseline $CD4^+/\mu l$	Baseline plVL[B]
4	LPV AZT 3TC	8	54	349	11053
17	LPV AZT 3TC	6	213	291	457275
22	LPV AZT 3TC	6	65	492	11000
23	LPV AZT 3TC	8	77	950	9150
25	LPV AZT 3TC	6	87	625	412857
38	LPV AZT 3TC	11	64	429	27800
41	LPV AZT 3TC / EFV AZT 3TC / TNV LPV DDI / TNV LPV 3TC	4	87	337	3620000
56	LPV AZT 3TC / EFV AZT 3TC / TNV EFV 3TC	5	70	724	495500
60	LPV AZT 3TC	6	55	245	422000
67	LPV AZT 3TC	12	71	310	114000
72	LPV AZT 3TC	7	75	564	56300
73	LPV AZT 3TC	7	84	411	132000
76	LPV AZT 3TC	4	22	401	12200000
78	LPV AZT 3TC / RTV AZT ATV 3TC	3	76	351	1360000
81	LPV AZT 3TC / RTV AZT ATV 3TC / TNV ETC EFV / TNV LPV ETC	6	92	ND	33150000
92	LPV AZT 3TC	4	106	228	6050000
97	LPV AZT 3TC	13	51	656	14400
99	LPV AZT 3TC / TNV RTV ETC ATV	5	74	608	75200
102	LPV AZT 3TC	6	66	196	653000
112	LPV AZT 3TC	5	66	226	29300000
114	LPV AZT 3TC / AZT 3TC / TNV RTV ETC ATV	5	47	502	475000
129	LPV AZT 3TC	4	55	493	1090000
133	LPV AZT 3TC / TNV LPV ETC / TNV RTV ETC ATV	15	52	683	81700
143	LPV AZT 3TC / TNV LPV ETC	7	59	170	428000
	Range (min/max)	3/15	22/213	170/950	$9.2x10^3/3.3x10^7$
	Percentiles (25%/75%)	5/8	55/82	291/608	$6.1x10^4/1.3x10^6$
	Median	6	68	411	4.3×10^5

[A] Antiretroviral drugs subsequently used: 3TC=Lamivudine, ATV=Atazanavir, AZT=Zidovudine, D4T=Stavudine, DDI=Didanosine, EFV=Efavirenz, ETC=Emtricitabin, LPV=Lopinavir, NFV=Nelfinavir, RTV=Ritonavir, SQV=Saquinavir, TNV=Tenofovir,
[B] HIV-1 RNA copies/ml in plasma as measured with the Roche®-Taqman test.

TABLE S1-B: Characteristics of patients in the *"chronic"* group

ID[A]	Treatments)[B]	Duration of cART (weeks)	Baseline $CD4^+$/µl	cART $CD4^+$/µl	Baseline pIVL[C]	cART pIVL[C]
ts101	LPV AZT 3TC	37	312	695	86200	11
ts102	LPV AZT 3TC	43	292	306	22700	0
ts105	LPV AZT 3TC	48	126	251	114000	28
ts106	LPV AZT 3TC	55	164	402	47700	0
ts108	LPV AZT 3TC/LPV D4T 3TC/LPV AZT 3TC	50	7	41	184500	13
ts109	TNV LPV 3TC	38	142	206	334500	6
ts110	LPV AZT 3TC	48	200	432	186000	0
ts111	LPV AZT 3TC/TNV LPV DDI	36	116	66	14300	0
ts112	LPV AZT 3TC	36	121	368	158000	7
ss102	AZT 3TC/IDV AZT 3TC	183	140	723	561831	0
ss103	NFV DDI D4T	76	181	318	26568	25
ss104	AZT 3TC/IDV AZT 3TC/NFV AZT 3TC	131	252	515	1502850	25
ss108	DDI AZT/AZT/RTV AZT 3TC	152	143	348	nd	0
ss110	NFV DDI D4T	88	121	508	58175	21
ss119	SQV RTV DDI D4T/SQVH RTV D4T 3TC	79	242	440	113052	18
	Range (min/max)	36/183	7/312	41/723	$1.4x10^4/1.5x10^6$	0/28
	Percentiles (25%/75%)	38/88	121/242	251/508	$4.2x10^4/2.2x10^5$	0/21
	Median	50	143	368	$1.1\ x10^5$	7

[A] Patient No: ts: patients from The Zurich HIV-1 Transcription Study (INFZ VTA 02.00) [12], ss: patients from the SSITT trial [33].
[B] Antiretroviral drugs subsequently used: 3TC=Lamivudine, ATV=Atazanavir, AZT=Zidovudine, D4T=Stavudine, DDI=Didanosine, EFV=Efavirenz, ETC=Emtricitabin, LPV=Lopinavir, NFV=Nelfinavir, RTV=Ritonavir, SQV=Saquinavir, TNV=Tenofovir.
[C] HIV-1 RNA copies/ml in plasma as measured with the Roche®-Amplicor test.
