# Supplementary material for: Profound Depletion of HIV-1 Transcription in Patients Initiating Antiretroviral Therapy during Acute Infection
Source: PLoS One. 2010 Oct 12;5(10):e13310. doi: 10.1371/journal.pone.0013310 (PMC2953504; doi:10.1371/journal.pone.0013310)
Supplement: Table S2 — Oligoucleotides used for patient-specific qPCR. (0.10 MB PDF) [file pone.0013310.s002.pdf]

Table S2-A: Oligoucleotides used for patient-specific qPCR – UsRNA, vRex, vDNA

ID [A]	Function (Polarity) [B]	Position [C] 5'-3'	Sequence [D]	Patient-ID [E]
mf302	Antisense (-)	2634-2662	CAAATTTCTACTAATGCTTTTATTTTTT	**56**, **17**, **22**, **25**, **38**, **60**, **76**, **92**, **99**, **102**, **112**, **114**, **129**, **4**, ts101, ts108, ts102, ts111, ss103, ss104, ss110
			CATATTTCTGTTAAAGCTTTTATTTTTTC	**72**
			CAAATTTCTGCTAATGCTTTTATCTTTTC	**41**
			CAAATTTCTACTAAGGCTTTTACCTTTTC	**67**
			CATATTCCTACTAATGCTTTTATTTTTTC	**73**, **78**
			CATATTTCTACTAATGCTTTTATTTTTTC	**133**
			CAAATTTCTACTAGTGCTTTTATTTTCTC	**23**
			CAAATTTCTATTAATGCTTTTATTTTTTC	**81**, ts112
			CAAATTTCTACTAATGCTCTTATTTTTTC	**97**, **143**
			CAAATTTCTACTAATGCTTTTATCTTTTC	ss102
			CAGATTTCTACTAATGCTTTTATTTTTTC	ss108
			CAGATTTCTGTTAATGCTTTTATTTTTTC	ts110
			CAAATTTCTGTTAATGCTTTTATTTTTTC	ts109
			CAAATTTCTACTAGTGCTTTTATTTTTTC	ts105, ss119
mf299	Sense (+)	2536-2562	GCACTTTAAATTTTCCCATTAGTCCTA	**41**, **17**, **22**, **25**, **38**, **60**, **76**, **81**, **92**, **112**, **129**, **143**, **4**, ts108, ts112, ts102, ts105, ts111, ss103, ss104, ss108, ss110
			GTACTTTAAATTTTCCAATAAGTCCTA	**72**
			GCACTTTAAATTTTCCTATTAGTCCTA	**56**
			GCACTTTAAATTTCCCTATTAGCCCTA	**67**
			GTACTTTAAATTTTCCCATTAGTCCTA	**73**, **78**, **133**, **114**
			GCACTCTAAATTTTCCCATTAGTCCTA	**23**, **97**, **99**, ts110, ss119, ss102,
			GCACTTTAAATTTTCCAATTAGTCCTA	**102**
			GCACTTTAAATTTTCCCATAAGTCCTA	ts101
			GTACTTTAAATTTCCCAATTAGTCATA	ts109
mf304	Probe (+)	2599-2624	f-ATGGCCCAAAAGTTAAACAATGGCCA-q	**67**, **38**, **60**, **76**, **97**, **99**, **25**, ss103
mf309	Probe (+)	2611-2633	f-TTAAACAATGGCCATTGACAGAA-q	**72**, **41**, **56**, **73**, **78**, **133**, **17**, **23**, **4**, ts105
mf348	Probe (+)	2586-2604	f-AAGCCAGGAATGGATGGCC-q	**81**, **92**, **102**, **112**, **114**, **129**, **143**, ts101, ts108, ts110, ts109, ts112, ts102, ss119, ss102, ss108,
ri16	Probe (+)	2619-2631	f-CTG**Ť**C**Ă**A**Ť**A**Ğ**CCA-q	**22**, ts111, ss104, ss110

[A] ID, oligonculeotide-designation of wild-type sequence (HXB2, GenBank accession number K03455)
[B] Function; sense - amplification primer of + polarity, antisense- amplification primer of – polarity, probe, fluorescent-hydrolysis (Taqman®) probe.
[C] Position of the wild-type sequence (+ polarity) in the HXB2 sequence according to the Los Alamos HIV database (www.hiv.lanl.gov)
[D] Sequences are indicated 5' to 3'. Underlined, deviation from standard sequence. Base modifications, **Č, Ğ, Ă, Ť,** show positions of locked nucleic acid base analogues. Fluorescein moiety attached to the 5' termini of fluorescent-probes (f-), quencher (TAMRA) attached to the 3' termini of fluorescent-probes (-q).
[E] Numbers in bold are patients from the Zurich Primary HIV infection study (acutely infected) and plain numbers are referring to chronically infected patients.

Table S2-B: Oligoucleotides used for patient-specific qPCR – MsRNA-tatrev

ID [A]	Function (Polarity) [B]	Position [C] 5'-3'	Sequence [D]	Patient-ID
mf83	Antisense (-)	8459-8433	GGATCTGTCTCTGTCTCTCTCTCCACC	**38**, **81**, **129**, **112**
			GGATCTGTCTCTGCCTCTCTCTCCACC	**60**
			GGATGTGTCTCTGTCTCTCTCTCCACC	**76**, **17**
			GGATCTGCCTCTGTCTCTCTCTCCACC	**92**
			GGATATGTCTCTGTCTCTCTCTCCACC	**97**
			GGATCTGTCTCTGTCTTGCTCTCCACC	**99**, **22**
			GGATCTGTCTCTGTCTTTCTCTCCACC	**102**
			GGATCTGTTGCTGTTTCTTTCTCCACC	**114**
			GGATGAATCTCTGCCTCTCTCTCCACC	**143**
			GGATCTGTCTCTGCCTCGCTCTCCACC	**4**
			GGATCTGGTTCTGTCTCTCTCTCCACC	**41**
			GGATCTGTCTCTGTCTCTGTCTCCACC	**56**
			GGATGTGTCTCTCTCTCTCTCTCCACC	**67**
			GGATGTGTCTCTGTCTCTCCCTCCACC	**73**, **78**, **133**
			TGATCTGTCTCTGTCTTGCTCTCCACC	**23**
			GGATCTGTCTCTGTCTGTCTCTCCACC	**72**, **25**
mf1b	Sense (+)	5956-5978	CTTAGGCATCTCCTATGGCAGGA	**38**, **41**, **56**, **67**, **72**, **76**, **81**, **92**, **102**, **112**, **129**, **143**, **4**, **17**, **23**, **25**
			CTTAGGCATCTCATATGGCAGGAA	**73**, **78**, **133**, **22**
			CTTAGGCGTCTCCTATGGCAGGA	**60**
			CTTAGGCATCTCCCATGGCAGGA	**97**
			CTTAGGCATCTTCTATGGCAGGAA	**99**
			CTTAGGCATTTACTATGGCAGGA	**114**
mf226tq	Probe (+)	8397-8414	f-AGGGGACCCGACAGGCCC-q	**38**, **60**, **92**, **97**, **102**, **112**, **114**, **129**, **143**
as1tq	Probe (+)	5979-5996	f-AGAAGCGGAGACAGCGAC-q	**25**, **23**, **41**, **56**, **67**, **73**, **78**, **133**, **17**, **22**, **76**, **81**, **4**, **72**
ri13	Probe (+)	8405-8417	f-CGA**Č**A**Ğ**G**Č**CC**Ğ**AA-q	**99**

[A] ID, oligonculeotide-designation of wild-type sequence (HXB2, GenBank accession number K03455)
[B] Function; sense - amplification primer of + polarity, antisense- amplification primer of – polarity, probe, fluorescent-hydrolysis (Taqman®) probe.
[C] Position of the wild-type sequence (+ polarity) in the HXB2 sequence according to the Los Alamos HIV database (www.hiv.lanl.gov)
[D] Sequences are indicated 5' to 3'. Underlined, deviation from standard sequence. Base modifications, **Č, Ğ, Ă, Ť,** show positions of locked nucleic acid base analogues. Fluorescein moiety attached to the 5' termini of fluorescent-probes (f-), quencher (TAMRA) attached to the 3' termini of fluorescent-probes (-q).

Table S2-C: Oligoucleotides used for patient-specific qPCR – MsRNA-total

ID [A]	Function (Polarity) [B]	Position [C] 5'-3'	Sequence [D]	Patient-ID
mf83	Antisense (-)	8459-8433	GGATCTGTCTCTGTCTCTCTCTCCACC	**38**, **81** , **129**, **112**
			GGATCTGTCTCTGCCTCTCTCTCCACC	**60**
			GGATGTGTCTCTGTCTCTCTCTCCACC	**76**, **17**
			GGATCTGCCTCTGTCTCTCTCTCCACC	**92**
			GGATATGTCTCTGTCTCTCTCTCCACC	**97**
			GGATCTGTCTCTGTCTTGCTCTCCACC	**99**, **22**
			GGATCTGTCTCTGTCTTTCTCTCCACC	**102**
			GGATCTGTTGCTGTTTCTTTCTCCACC	**114**
			GGATGAATCTCTGCCTCTCTCTCCACC	**143**
			GGATCTGTCTCTGCCTCGCTCTCCACC	**4**
			GGATCTGGTTCTGTCTCTCTCTCCACC	**41**
			GGATCTGTCTCTGTCTCTGTCTCCACC	**56**
			GGATGTGTCTCTCTCTCTCTCTCCACC	**67**
			GGATGTGTCTCTGTCTCTCCCTCCACC	**73**, **78**, **133**
			TGATCTGTCTCTGTCTTGCTCTCCACC	**23**
			GGATCTGTCTCTGTCTGTCTCTCCACC	**72**, **25**
mf84	Sense (+)	6012-6045	ACAGTCAGACTCATCAAGTTTCTCTATCAAAGCA	
			CAGTGAGACTGATCAAGCTTCTCTATCAAAGCA	**41**
			CAGTCAGACTCATCAGGTTTCTCTATCAAAGCA	**56**
			CAGTCAGAATCATCAGGATTCTCCATCAAAGCA	**67**
			CCGTCAGAATCATCAAGTTCCTCTACCAAAGCA	**73**
			CCGTCAGAATCATCAAGATCCTCTACCAAAGCA	**78**
			CCGTCAGACTCATCAAGTTCCTCTACCAAAGCA	**133**
			CCGTGAGACTCATCAAGCTTCTCTATCAAAGCA	**17**
			CAGTCAGACTCATCAAGTTCCTCTACCAAAGCA	**22**
			CAGTACGCCTCATCAGGTCGCTCTATCAAAGCA	**23**
			CAGTCAGACTGATCAAGTTTCTCTATCAAAGCA	**25**
			ACAGTGAGACTGATCAAAGTTCTCTATCAAAGCA	**38**
			ACAGTCAGACTCATCAAGCTTCTCTATCAAAGCA	**60**, **99**, **102**
			ACAGCCAGACTCATCAGGATTCTCTATCAAAGCA	**76**
			ACCGTCAGGCTAATCAAGTTTCTCTACCAAAGCA	**81**
			GCAGTCAGACTCATCAAGTTTCTCTATCAAAGCA	**92**
			GCAGTCAGGCTCATCAAGTTTCTCTACCAAAGCA	**97**
			ACAGTAAGACTCATCAAAAGACTCTATCAAAGCA	**112**
			ACAGTCAGACTCATCAAGATTCTCTACCAAAGCA	**114**
			ACGGTCAGACTCATCAGGTTTCTCTATCAAAGCA	**129**
			ACAGTCAGACTGATCAAGCTTCTCTATCAAAGCA	**143**
			ACAATCAGAATTATCAAGTTTCTCTATCAAAGCA	**4**
			CAGACTCATCAAGACTCTCTATCAAAGCA	**72**
mf226tq	Probe(+)	8397-8414	f-AGGGGACCCGACAGGCCC-q	**73**, **78**, **114**, **133**, **143**, **22**, **38**, **92**, **97**, **102**, **112**, **129**, **17**, **60**
			f-AGGGGACCCGACAGGCCA-q	**41**
			f-AGGGAACCCGACAGGCCA-q	**4**
mf2tq	Probe (+)	8421-8399	f-TTCCTTCGGGCCTGTCGGGTCCC-q	**67**, **76**
ri12	Probe (+)	8403-8415	f-CCC**Ğ**AC**Ă**G**Ğ**C**Č**CG-q	**72**, **81**
ri13	Probe (+)	8405-8417	f-CGA**Č**A**Ğ**G**Č**CC**Ğ**AA-q	**56**, **23**, **25**, **99**

[A] ID, oligonculeotide-designation of wild-type sequence (HXB2, GenBank accession number K03455)
[B] Function; sense - amplification primer of + polarity, antisense- amplification primer of – polarity, probe, fluorescent-hydrolysis (Taqman®) probe.
[C] Position of the wild-type sequence (+ polarity) in the HXB2 sequence according to the Los Alamos HIV database (www.hiv.lanl.gov)
[D] Sequences are indicated 5' to 3'. Underlined, deviation from standard sequence. Base modifications, **Č, Ğ, Ă, Ť,** show positions of locked nucleic acid base analogues. Fluorescein moiety attached to the 5' termini of fluorescent-probes (f-), quencher (TAMRA) attached to the 3' termini of fluorescent-probes (-q).
