# Supplementary material for: Profound Depletion of HIV-1 Transcription in Patients Initiating Antiretroviral Therapy during Acute Infection
Source: PLoS One. 2010 Oct 12;5(10):e13310. doi: 10.1371/journal.pone.0013310 (PMC2953504; doi:10.1371/journal.pone.0013310)
Supplement: Methods S1 — Supplementary methods. (0.05 MB PDF) [file pone.0013310.s003.pdf]

Methods S1: Supporting Information

S-1. Sampling

In total 539 plasma samples were analyzed, 22.4±1.8 (mean±sem) samples per patient:

– 0-24 weeks within cART initiation including baseline; n-total=129, 5.4±0.2 samples per patient.

– >24 weeks after cART initiation; n=147, 6.1±1.1 samples per patient.

– ≤24 weeks postcessation; n= 92, 3.8 ±0.3 samples per patient.

– >24 weeks postcessation; n=171; 7.1 ±1.0 samples per patient.

In total 257 PBMC samples were analyzed for unspliced HIV-1 (UsRNA), multiply spliced viral RNAs (MsRNAs) and HIV-1 total DNA (vDNA), 10.7±0.4 (mean±sem) samples per patient:

– 0-24 weeks within cART initiation including baseline; n=96, 4.0±0.2 samples per patient.

– >24 weeks after cART initiation; n=46, 2.1±0.2 samples per patient.

– ≤24 weeks postcessation; n= 65, mean samples per patient: 2.7 ±0.2 samples per patient.

– >24 weeks postcessation; n=50; mean samples per patient 2.3 ±0.2 samples per patient.

Viral RNA extracellularly bound to PBMC (vRex) [1,2,3,4] a parameter documented to be associated with ongoing virion production in PBMC, was assessed less frequently due to its anticipated lack of expression under cART in previous studies. Thus, 131 PBMC samples were analyzed for vRex, 5.5±0.1 (mean±sem) samples per patient:

– 0-24 weeks within cART initiation including baseline; n=56; 2.3±0.1 samples per patient.

– >24 weeks after cART initiation; n=6; 0.25±0.10 samples per patient.

– ≤24 weeks postcessation: n= 38; mean samples per patient: 1.6 ±0.1 samples per patient.

>24 weeks postcessation: n=31; mean samples per patient 1.3 ±0.2 samples per patient.

S-2. Primers and probes for HIV-1 qPCR

Patient virus matched variants of the following primers and probes were used for qPCR of HIV-1 nucleic acids as listed in detail in Supplement 3. Mf83 [4,5] was used antisense/cDNA primer for the quantification of both MsRNA-total and MsRNA-tatrev. MsRNA-total was amplified by sense primer mf84 [4]. MsRNA-tatrev was amplified by sense primer mf1b

(HXB2 sequence: CTTAGGCATCTCCTATGGCAGGA). For PCR in *pol* (UsRNA, vDNA, vRex), antisense/cDNA primer mf302 and sense primer mf299 were used [5].

To minimize complexity and cost of designing and synthesizing a multitude of individually matched fluorescent probes, a collection of highly conserved fluorescent probes with optimized performance was used [5] . Three probes were used to monitor amplification of MsRNA-tatrev, as1 (FAM-AGAAGCGGAGACAGCGAC-TAMRA), ri13 [5] and mf226 [2,5].

Four probes were used for MsRNA-total: mf226 [2,5], mf2tq, ri12 and ri14 [5]. Amplification in *pol* was monitored with 4 probes: ri16, mf348, mf304 and mf309 [5].

S-3. Primers for and probes for qPCR of cellular targets

GAPDH-RNA PCR was performed as previously described using primer mf45 for the sense strand, antisense/cDNA primer mf46 and, as a probe, mf70tq [6].

Beta-actin DNA was quantified using primers mf140 (CTCCCCCATGCCATCCTGCGTCTG) and mf141 (CTCGGCCGTGGTGGTGAAGC) and mf143tq (FAM-ACCTGGCTGGCCGGGA-TAMRA) as a probe.

S-4 Determination of intercepts and slopes for HIV-1 qPCR

Intercepts (*I)* were determined individually for each PCR and patient by determination of the mean *Ct* at or beyond the 50% dilution endpoint (samples showing ≥50% of replicates PCR-negative) resulting in the following mean (±sem) values for *I*; MsRNA-total: 43.0(±0.3), MsRNA-tatrev: 41.4(±0.3), UsRNA: 42.6(±0.4), vRex: 41.5(±0.53), vDNA: 43.1(±0.4). Since HIV-RNAs were often present at low levels, slopes could not be calculated for all patients. Therefore, the mean of acceptable slopes (≥3 PCR-positive dilutions before the 50% end-point; *S*<-3.0) were used for HIV-1 RNA calculation resulting in the following mean (±sem, n) values for *S*; MsRNA-total: -3.64(±0.04, n=8), MsRNA-tatrev: -3.32(±0.12, n=4), UsRNA: -3.30(±0.06, n=8), vRex: 3.42(±0.08, n=12). Due to the consistently high copy numbers in vDNA standards taken at baseline, slopes were determined individually (mean±sem of all slopes=-3.5±0.06). Using a larger dataset of acute patients (n=69) which includes the data from the present study was further analyzed to distribution and range of slopes and intercepts were further analyzed. In 17 patient-DNAs were monitored with HXB-2 primers (wt) and in 57 patients with 1 or 2 non-HXB-2 sequence-adjusted primers (sa). Only a trend

in differences in slopes (mean±sem, S_{wt}=-3.40±0.06, S_{sa}=-3.54±0.04, t-test p=0.07) and no difference in intercepts (I_{wt}= 42.11±0.65, I_{sa}= 42.26 ± 0.26, t-test p=0.81) were observed. These results further substantiate the validity of our patient matched approach to qPCR.

References

1. Fischer M, Joos B, Niederost B, Kaiser P, Hafner R, et al. (2008) Biphasic decay kinetics suggest progressive slowing in turnover of latently HIV-1 infected cells during antiretroviral therapy. Retrovirology 5: 107.
2. Kaiser P, Joos B, Niederoest B, Weber R, Günthard HF, et al. (2007) Productive Human Immunodeficiency Virus 1 Infection in peripheral Blood Predominantely Takes Place in CD4/CD8 double negative T Lymphocytes. J Virol 81: 9693-9706.
3. Fischer M, Joos B, Wong JK, Ott P, Opravil M, et al. (2004) Attenuated and nonproductive viral transcription in lymphatic tissue of human immunodeficiency virus type 1 infected patients on potent antiretroviral therapy. JInfectDis 189: 273-285.
4. Fischer M, Joos B, Hirschel B, Bleiber G, Weber R, et al. (2004) Cellular viral rebound after cessation of potent antiretroviral therapy predicted by levels of multiply spliced HIV-1 RNA encoding nef. J Infect Dis 190: 1979-1988.
5. Althaus CF, Gianella S, Rieder P, von Wyl V, Kouyos RD, et al. (2010) Rational design of HIV-1 fluorescent hydrolysis probes considering phylogenetic variation and probe performance. J Virol Methods 165: 151-160.
6. Roscic-Mrkic B, Fischer M, Leemann C, Manrique A, Gordon CJ, et al. (2003) RANTES (CCL5) utilizes the proteoglycan CD44 as an auxiliary receptor to mediate cellular activation signals and HIV-1 enhancement. Blood 102: 1169-1177.
